# Post-Translational Modifications in Respiratory Virus Infection: Recent Insights into the Development of In Vitro Models

**DOI:** 10.3390/ijms262412174

**Published:** 2025-12-18

**Authors:** Emna Ben Khlifa, Alessia Campese, Andrea Corsi, Cristina Bombieri, Maria Grazia Romanelli, Maria Teresa Valenti, Donato Zipeto, Matteo Castelli, Patricia Marie-Jeanne Lievens, Alessandra Ruggiero

**Affiliations:** 1 Department of Neurosciences, Biomedicine, and Movement Sciences, School of Medicine, University of Verona, Strada le Grazie 8, 37134 Verona, Italy; emna.benkhlifa@univr.it (E.B.K.); cristina.bombieri@univr.it (C.B.); mariagrazia.romanelli@univr.it (M.G.R.); mariateresa.valenti@univr.it (M.T.V.); donato.zipeto@univr.it (D.Z.); patricia.lievens@univr.it (P.M.-J.L.); 2 Laboratory of Microbiology and Virology, Vita-Salute San Raffaele University, 20132 Milan, Italy

**Keywords:** post-translational modifications, emerging respiratory viruses, virus–host interaction, virus infection, organoids

## Abstract

Post-translational modifications (PTMs) are crucial chemical alterations occurring on proteins post-synthesis, impacting various cellular processes. During viral infections, PTMs are shown to play a multitude of roles in viral replication, host interaction, and immune evasion. Thus, these modifications can influence infectivity, with direct impact on the anti-viral host immune responses and potentially viral adaptation across species. This field is still scarcely explored, whilst understanding PTMs is not only important to advance the knowledge of virus pathology but also potentially to provide insights for vaccine development. In this review, we attempt to summarize the latest findings mainly published over the last 10 years, focusing on the roles of PTMs involved in virus infection and anti-viral immune responses, in the context of relevant human respiratory infections: influenza A virus (IAV), respiratory syncytial virus (RSV), and SARS-CoV-2. We decided to concentrate on these three viruses because they currently represent a global health problem due to recurrent outbreaks and pandemic potential. A deeper characterization of the PTMs may help in understanding virus–host interaction with possible implications on curative strategies. Further, we will report on cutting-edge technologies to study in vitro virus infection in different cellular-based systems. In particular, we describe and discuss the application of 2D and 3D lung organoid cell-culture systems as in vitro models to mimic respiratory environments and to study the PTMs in a controlled setting. Finally, we will discuss the importance of PTMs in the context of next-generation vaccine design, especially for their potential role to offer effective protection against respiratory viruses.

## 1. Introduction

Post-translational modifications (PTMs) are catalyzed by specific enzymes and represent essential biochemical processes that modify proteins after synthesis, influencing their behaviour and function [1,2]. By introducing new functional biochemical groups, PTMs can alter protein stability, localization, interactions, and activity, enabling proteins to perform diverse roles within cellular pathways and responses to stimuli [3,4,5]. PTMs enhance the complexity of protein repertoire in eukaryotic cells by playing relevant roles in cellular homeostasis and signal integration [6,7], ultimately controlling organism health. When a virus infects its host, it faces a complex scenario of cellular processes, including PTMs, which can also influence the anti-viral immune response and survival of the infected organism [8].

In this review, we report the existing literature discussing the roles of PTMs in virus infection, with a specific focus on respiratory viruses: influenza A (IAV), respiratory syncytial virus (RSV), and SARS-CoV-2. We start by shortly describing the major biochemical and biological aspects of the most common PTMs, followed by a brief description of the infection mechanisms of IAV, RSV, and SARS-CoV-2, trying to highlight the PTMs involved. We then describe and discuss the potentiality and limitations of cutting-edge experimental models, such as organoids, available to study the role of specific PTMs in viral respiratory infections. In the discussion, we also briefly discuss how the presence of specific PTMs on viral proteins could impact the development of vaccines.

## 2. Post-Translational Modifications: A Strategy to Increase Protein Repertoire and Functionality

Most common PTMs include glycosylation, sulfation, phosphorylation, ubiquitination, methylation, acetylation, and lipidation.

Glycosylation occurs through the co- or post-translational attachment of carbohydrate moieties to asparagine, serine, or threonine residues, and it involves several endoplasmic reticulum and Golgi apparatus enzymes that catalyze the covalent attachment and remodelling of carbohydrate moieties to proteins. When carbohydrates are attached to asparagine residues, this is called N-linked glycosylation, whereas attachment to serine or threonine residues is called O-linked glycosylation. Glycosylation has been described in different biological processes, including protein folding and stability, cell adhesion [9,10,11], and modulation of membrane receptor activity. In the latter case, glycosylation may play direct roles in receptor transport and ligand binding, thus influencing both receptor activation and function [12,13].

Sulfation refers to the addition of a sulphate (SO_3_-) group to tyrosine, serine, or threonine amino acids catalyzed by specific sulfotransferases, often targeting membrane and secretory proteins. Interestingly, N-glycosylated proteins can also be sulfated via the transfer of a sulphate group from 3′-phoshoadenosine-5′ phosphosulfate (PAPS) onto Gal, GlcNAc, or GalNac sugar moieties, thus increasing glycosylation heterogeneity [14].

Phosphorylation is defined as the addition of a phosphate group to the hydroxyl group of serine, threonine, or tyrosine amino acids, which can alter the conformation, charge, and function of the protein [15,16]. The phosphate group is covalently attached to the amino acid residue via a phosphodiester bond, catalyzed by a kinase enzyme, while phosphatases catalyze the reverse reaction [17,18]. The turnover of the phosphor group is often quick, rendering this modification only transient compared to other PTMs. Phosphorylation is essential for several biological activities, including cell signalling, cell cycle regulation, apoptosis, and metabolism [19]. Dysregulation of phosphorylation can cause diseases such as neurodegenerative disorders [20], diabetes [21], bone diseases [22] and cancers [23], among others.

Ubiquitination and SUMOylation are the attachment of ubiquitin or small ubiquitin-like modifiers (SUMO) to lysine residues, respectively. Ubiquitination commonly marks proteins for degradation via the proteasome, thus regulating protein turnover and maintaining cellular homeostasis. This modification, involving the attachment of ubiquitin, a small protein consisting of 76 amino acids, to a target protein, is crucial for various biological processes such as cell signalling, programmed cell death, stress response, and DNA repair mechanisms [24]. In contrast, protein SUMOylation does not primarily lead to degradation. Instead, SUMOylation regulates the function and activity of various proteins, influencing processes like nuclear transport, transcriptional regulation, protein stability, DNA replication, and repair [25].

Methylation involves the addition of methyl groups to lysine or arginine residues in proteins, catalyzed by methyltransferase enzymes [26]. This modification, which can occur as mono-, di-, or tri-methylation, plays crucial roles in signal transduction, gene expression, and cell cycle regulation, among others [27].

Acetylation is a common post-translational modification found in both prokaryotic and eukaryotic organisms. It involves the transfer and addition of acetyl groups to proteins’ N-terminal amino group or lysine residues by either enzymatic or non-enzymatic processes, with the latter being more frequent. It can occur reversibly on the side chain of the lysine residue or irreversibly on the ε-amino group at the N-terminus. N acetyltransferase (NAT) catalyzes most of the N-terminal acetylation modification on eukaryotic cells, while lysine acetylase (KAT) and lysine deacetylase (KDAC) mostly catalyze lysine reversible acetylation [28]. This kind of modification is associated with various cellular processes and enzymatic activities, such as providing stability to DNA, protein–protein interactions, and DNA binding [29].

Protein lipidation is a co- or post-translational protein modification in which lipid moieties are covalently attached to target proteins [30]. Major protein lipidations include S-palmitoylation, N-myristylation, and S-prenylation, all playing relevant physiological functions [31]. Among protein lipidations, S-palmitoylation has the unique feature of being reversible and, therefore, undergoes cellular regulation. S-palmitoylation occurs when a thioester bond serves to covalently link the 16-carbon saturated fatty acid palmitic acid to specific cysteine residues in proteins. The reaction is catalyzed by one of the 23 different mammalian ZDHHC palmitoyl transferases discovered [32,33,34] and can be reversed by palmitoyl thioesterases. Interestingly, tyrosine phosphorylation can regulate S-palmitoylation catalysis, implicating a functional interplay between different PTMs [35]. S-palmitoylation renders the substrate more hydrophobic, and it is thus frequently found on signalling proteins that need to interact with cell membranes, as in the case of several members of the MAPK pathways [36]. Dysregulation of S-palmitoylation has been associated with several diseases [31].

These PTMs can profoundly influence the properties and functions of proteins [8], and viruses frequently take advantage of the cellular machinery to alter viral and host PTMome, which improves their replication and survival while evading host immune defences [37]. In this context, glycosylation and polysaccharide sulfation have been proven to enhance humoral and cell-mediated immune response [38]; on the other hand, Zarling [39] and colleagues showed that cytotoxic T lymphocytes (CTLs) can recognize phosphopeptides, leading to an immune response, but not non-phosphorylated ones. Recently, SUMOylation was shown to play a crucial role in both innate and adaptive immunologic responses, as well as pathogen-host immune system crosstalk [40] and one study reported that HIV-1 accesses the host acetylation network to promote infection at multiple steps during the viral life cycle [41]. Moreover, S-palmitoylation has been recently shown to play key roles in host–virus interactions during infection of IAV [42], RSV [43], SARS-CoV-2 [44,45,46].

## 3. Respiratory Viruses Infection Mechanisms and PTMs

Respiratory viruses are currently under intense investigation, because of the recent pandemic or potential for pandemic, alongside raising concerns about clinical outcomes associated with seasonal endemic circulation. Despite still being poorly described, PTM’s role in the respiratory viruses’ infection cycle and pathogenesis has been reported by some groups and are summarized in Table 1.

**Figure 1:**
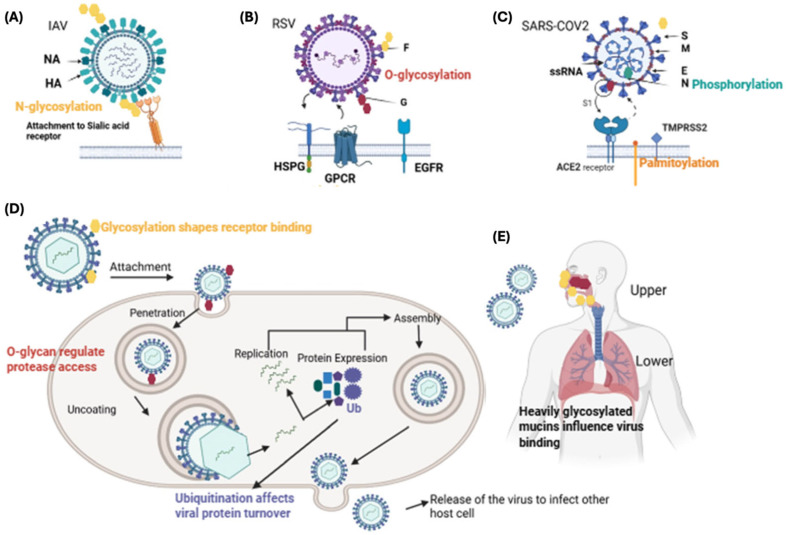
**Viral surface post-translational modifications (PTMs) and their roles during infection.** (**A**) Influenza A virus (IAV). The IAV envelope contains hemagglutinin (HA) and neuraminidase (NA), both extensively N-glycosylated, which contribute to proper protein folding, immune evasion, and modulation of receptor binding. HA binds to sialic acid receptors on host epithelial cells to mediate entry. (**B**) Respiratory syncytial virus (RSV). RSV expresses the F and G glycoproteins on its surface. The G protein is rich in O-linked glycans, which influence host immune recognition and interaction with several cellular receptors, including heparan sulphate proteoglycans (HSPG), G-protein–coupled receptors (GPCRs), and the epidermal growth factor receptor (EGFR). (**C**) SARS-CoV-2. The viral surface spike (S) protein carries multiple N-glycans that help shield antigenic epitopes and regulate interaction with the ACE2 receptor. The nucleocapsid (N) protein undergoes phosphorylation, which supports viral RNA packaging and replication. The cytosolic tails of S and other structural proteins (E and M) can undergo palmitoylation, promoting virion assembly and membrane association. Activation of the S protein for cell entry is facilitated by TMPRSS2 protease at the plasma membrane. (**D**) PTM involvement during the viral life cycle. After attachment, PTMs regulate several intracellular steps: glycosylation patterns shape receptor affinity during entry; O-glycans near cleavage sites modulate protease accessibility for viral fusion; ubiquitination of viral proteins influences their stability, turnover, and the regulation of viral replication and assembly. The figure outlines the sequential stages of attachment, penetration, uncoating, replication, protein expression, assembly, and egress. (**E**) Virus interaction with the human respiratory tract. During transmission, respiratory viruses encounter heavily glycosylated mucins in the upper airway, which can modulate viral binding and tropism before the virus reaches the lower respiratory tract. NA, neuraminidase; HA, hemagglutinin; G, RSV G protein; F, RSV fusion protein; HSPG, heparan sulphate proteoglycan; GPCR, G-protein–coupled receptor; EGFR, epidermal growth factor receptor; S, spike; E, envelope; M, membrane; N, nucleocapsid; ssRNA, single-stranded RNA; ACE2, angiotensin-converting enzyme 2; TMPRSS2, transmembrane serine protease 2. Created with BioRender.com.

First, it must be noted that the pathogenicity and infectivity of these viruses vary greatly according to the body site they infect. The upper respiratory tract (URT) is the entry site, and most frequently the replication is restricted, while lower respiratory tract (LRT)—bronchi and lungs—involvement is a secondary and less frequent event (as depicted in Figure 1), generally associated with severe symptomatology.

The upper and lower tracts are populated by several cell types that express surface receptors with different affinities for respiratory viruses. This diversity in receptor expression plays a crucial role in determining a differential tropism for specific regions of the respiratory system, leading to distinct patterns of infection for each virus based on the types of cells they can bind to and invade. Considering the variety of receptors expressed in the upper and lower respiratory tract, PTMs could not only affect viral proteins, but also host factors influencing the infectivity and pathogenicity across different respiratory sites [47]. These modifications can either enhance or inhibit the virus’s ability to infect and cause disease, demonstrating the complex interaction between viral mechanisms and host defences. In the following paragraphs, we will focus on the PTMs involved in IAV, RSV, and SARS-CoV-2 infections.

### 3.1. IAV

IAV attaches to the surface of respiratory epithelial cells by binding to sialic acid through the hemagglutinin (HA). Upon entry, viral RNAs are imported into the nucleus, where transcription and replication take place. Assembly, budding, and release take place contextually at the plasma membrane, where the viral proteins HA, neuraminidase (NA), and M2 interact with the ribonucleoproteins (vRNPs) and M1. NA plays a crucial role during these processes by cleaving out sialic acid from the surface of infected cells and viral progeny to promote efficient release. Furthermore, several pieces of evidence support the NA role in the extracellular phases of the IAV life cycle, where sialic acid degradation in the mucus layer promotes virion access to the surface of epithelial cells [48]. IAV shows peculiar infection patterns according to the different sections of the respiratory tract that are largely determined by the preferential type of sialic acid expressed by epithelial cells and the specific tropism of IAV subtype/strains. Human IAV has a preferential tropism for the URT due to a higher affinity for sialic acid with an α2-6 linkage that is selectively expressed by nasal cavity and throat epithelial cells [49]. As described by the Centre for Disease Control (CDC) [50], as the infection progresses, IAV can move down to the trachea and bronchi, and in severe cases, it can infect the lung alveoli. Yet, for human IAV, this occurrence usually happens only in specific high-risk individuals due to the preferential expression of α2-3 sialic acid by LRT cells, towards which the virus has lower affinity. The clinical progression is worsened by the disproportionate inflammatory response that usually characterizes viral atypical pneumonia [49,51,52]. Worth mentioning, avian IAV has a marked preferentiality for α2-3 sialic acid—as it is highly expressed in birds’ enteric tracts—which results in a low risk of avian-to-human transmission but a high probability of developing severe atypical pneumonia when a human infection happens. Therefore, a PTM such as sialylation of host cell surface proteins and lipids is at the basis of IAV host tropism and physiopathology.

In the context of the IAV, different PTMs of viral proteins have been characterized to be involved in the virus’s life cycle and host immune response [47]. Glycosylation of the HA has been described to help the virus bind to host receptors and the evasion from the immune system, with variations in glycosylation affecting the virus’s ability to infect upper and lower tracts [53]. Furthermore, Li and colleagues [47] showed that, in the context of H5N1 HA, the N158 glycosylation can cause the dimerization of two HA trimers that impairs the exposure of epitopes important for the induction of neutralization antibodies, an aspect to be considered when developing anti-H5N1 vaccines. Phosphorylation of viral proteins has also been shown to regulate viral replication and assembly, thereby influencing the spread of the virus [47]. This modification affects the function of several viral proteins, including the NP and polymerase complex, which are essential for viral RNA synthesis, thereby influencing the efficiency of viral replication and the subsequent spread of the virus within the host. Other studies have shown that ubiquitination and SUMOylation can modulate viral stability and immune evasion [54,55,56]. About acylation, it has been observed that at least HA and M2 require S-palmitoylation for viral replication. Particularly, palmitoylation of HA promotes the assembly and release of virions from the host cell membrane, thereby influencing the infectivity of the virus [47,57].

### 3.2. RSV

Similar to IAV, RSV interacts with the host cell receptors through its attachment (G) and (to a lesser extent) fusion (F) proteins to infect respiratory epithelial cells. The viral RNA-dependent RNA polymerase replicates and transcribes the viral RNA in the cytoplasm once it has entered the cell. Major viral proteins, including the matrix (M) protein and the phosphoprotein (P), are phosphorylated to control progeny assembly taking place from the host cell membrane [58]. In adults, RSV typically manifests as upper respiratory tract infections with mild to moderate symptoms, only rarely causing severe disease [59], while in infants (particularly in the first two years of life), the elderly, and those with compromised immunity [59], RSV infection can cause bronchiolitis with a relevant incidence.

Depending on the infection site, the severity of RSV infection varies widely, and PTMs of viral proteins have been shown to substantially impact RSV infectivity. In the UTR, glycosylation of the G protein promotes viral attachment and entry into epithelial cells in the nasal cavity and throat [60] hence assisting initial infection and dissemination [61,62]. Furthermore, RSV can use the host ubiquitin–proteasome system to degrade host antiviral proteins, evading immune responses and increasing viral replication [63]. Moreover, it has recently been found that the oligomerization of the nucleoprotein is mediated by phosphorylation, and it is involved in the spatial and temporal regulation of viral genome encapsidation, which is critical for virus replication [64]. It has also been described that S-palmitoylation is important for viral particle formation and their infectivity, and for viral replication [43].

### 3.3. SARS-CoV-2

SARS-CoV-2 interacts with host cells via the spike (S) protein, which recognizes the angiotensin-converting enzyme 2 (ACE2) receptor, where glycosylation of the S protein is determinant for both host interaction and immune evasion [65]. It is worth noting that glycosylation of ACE2 can impact SARS-CoV-2 entry efficiency in a variant- and host-specific fashion [66,67]. Once the virus binds to the receptor, it undergoes membrane fusion, allowing its single-stranded RNA genome to enter the host cell. Following that, protein translation and replication of the viral RNA occur, leading to the assembly and release of new virions that infect other cells [68]. The viral nucleocapsid (N) protein has been shown to promote replication, while the viral membrane (M) plays a central role in virus assembly [65].

Similar to the other viruses, SARS-CoV-2 shows a spectrum of infectivity in different body regions, which results in a variety of clinical outcomes. Initially, the virus infects the URT, causing symptoms like fever, cough, and sore throat [69], and it can further replicate in the LRT, leading to worse symptomatology. Some PTMs of viral proteins have been identified as important players in this process. For instance, variation in glycosylation patterns can increase the capacity of the virus to replicate in certain locations, increase the stability of viral proteins, and increase the ability to interact with host receptors [36,70,71]. In the LTR, phosphorylation of viral proteins regulates viral replication and assembly and increases the virus’s ability to infect the bronchi and alveoli, leading to more serious illnesses such as pneumonia and acute respiratory distress syndrome (ARDS) [72]. It has also been shown that ubiquitination of the M protein can reduce viral replication [73]. Moreover, S-palmitoylation of the E protein has been shown to improve protein stability, thus enhancing the formation of viral particles [74]. Further, during the SARS-CoV-2 pandemic waves, several virus variants have been described to have different tropism for the upper and lower respiratory tract. For example, Delta SARS-CoV-2 variants appeared to be the most cytopathic for both upper and lower respiratory cells, whilst Omicron had improved replication in the upper-respiratory system [75]. These different behaviours have not been directly associated with PTMs, but further studies should explore this possibility. In short, described PTMs related to SARS-CoV-2 infections are phosphorylation, as found in the regulation of the N protein, glycosylation, as described for the S protein, but also S-palmitoylation that targets the M protein, promoting membrane fusion [76], and the envelop (E) protein plays a central role in particle formation [74].

It must be noted that PTMs can also affect the functions of host proteins implicated in the immune response, thereby altering signalling pathways, either enhancing viral replication or assisting the host in fighting the infection [77,78]. For example, ubiquitination and SUMOylation of host proteins implicated in immunological responses might promote viral persistence or cause excessive inflammation, increasing lung tissue damage [79,80].

These PTM-driven mechanisms emphasize the complicated interaction of viral approaches and host defences, which influences the severity and spread of virus infection across body sites. To deepen these aspects, in vitro models can be useful tools, and efforts are currently ongoing to optimize such systems.

## 4. In Vitro Systems to Model Respiratory Viruses’ Infection and PTMs

In vitro systems, by providing controlled environments for the evaluation of virus–host interactions, could represent useful tools for the study of respiratory viruses and PTMs. Various in vitro models ranging from simple cell monolayers to complex, three-dimensional (3D) cultures and organoids have already been used to understand viral pathogenesis.

The simplest form of an in vitro system involves epithelial cell lines that are either representative of the natural site of infection (e.g., A549 and Calu-3, both derived from human lung adenocarcinoma) or, in general, highly permissive to infection (e.g., Vero, derived from African green monkey kidney cells) [81]. These models are highly utilized to study virus replication cycles and screen antiviral drugs due to their accessibility and ease of use [81]. However, considering that they are cell lines, they do not have the capacity to offer a system that represents the complexity of human airway tissues [82]. Thus, to better mimic the human respiratory tract, primary Human Airway Epithelial Cells (HAE) can be grown in air–liquid interface (ALI) cultures, forming a pseudostratified layer with ciliated and goblet cells [83]. These models can simulate physiological conditions, resembling the human airway’s architecture. This aspect allows the study of virus entry, replication, and the evaluation of the cytopathic effects of the virus. Lung organoids and alveolar spheroids, containing different cell types (e.g., alveolar epithelial cells, fibroblasts, and immune cells), could represent another upgrade option to study respiratory viruses and PTMs, as we discuss in the following sections [82,84].

### Human Airway Organoids for In Vitro Modelling of the Respiratory System

As explained above, the human respiratory system is canonically divided into upper and lower airways. The great majority of 3D in vitro models focus on the lower airways, particularly lung tissues, but in recent years, a great surge of interest has arisen towards nasal organoids [2,82,85,86]. By definition, an organoid is a multicellular organ-specific structure developed from stem cells or progenitors, which is able to self-organize to resemble a tissue structure and composition upon differentiation [87,88]. In most cases, airway organoids can be produced from adult stem cells (ASCs) or pluripotent stem cells (PSCs) [82]. ASCs isolated from lung tissue possess self-renewal abilities, and their differentiation potential is limited to cell types normally present in lung tissues. On the other hand, PSCs, such as embryonic stem cells (ESCs) and induced pluripotent stem cells (iPSCs) are completely undifferentiated and need to be guided towards the desired cell type. This can be achieved in several ways. One approach involves the aggregation of undifferentiated PSCs into embryoid bodies (EBs) to further allow differentiation into the specific germ layers, mimicking embryonal gastrulation [89,90]. Another methodology involves inducing the spontaneous aggregation of cells during their differentiation into anterior foregut endoderm. Then, the differentiated clumps are embedded into an extracellular matrix to allow complete maturation [91].

## 5. Investigating Viral Infections Mechanisms and PTMs via 3D Organoids

Several studies explored the possible use of organoids in the context of respiratory viruses’ pathogenesis for IAV (Table 2), RSV (Table 3), and SARS-CoV-2 (Table 4).

While organoid models of SARS-CoV-2 infection have been developed for both the upper and lower airways, IAV organoid modelling is particularly focused on the lower airways. In fact, the principal organoid platforms used for disease modelling include lung organoids [93,96], lung bud organoids [108] and alveolar lung organoids [109]. For RSV, the most prominent in vitro models included lung organoids [90,97,98], lung bud organoids [99], and nasal organoids [85,97]. Disease modelling for RSV has an additional degree of complexity, given by the specific susceptibility of children to this infection [110,111]. In a recent work, Aloisio and colleagues generated nose organoids from pediatric and adult nasal swabs and nasal washes [85]. When subjecting the organoids to RSV infection, they observed increased cellular damage in the nasal organoids generated from children’s samples, mimicking what is observed in clinics [85].

In the context of PTMs analysis, the use of organoids faces several challenges [112]. First, organoids are embedded in protein-rich extracellular matrix (ECM), which could confound traditional liquid chromatography-tandem mass spectrometry approaches to study proteins with biochemical modifications. While it is technically possible to remove organoids from the ECM before this analysis, dissociation of live cells alters signalling pathways, causing a discrepancy between PTMs measurements from dissociated and non-dissociated organoids [112,113]. Additionally, the heterogeneous nature of organoids in terms of cell types and cell states represents a hard-to-overcome challenge for PTM-proteomics [114]. Finally, low-dimensional methodologies like fluorescent imaging cannot unravel the complexity of PTM-related signalling networks like high-throughput technologies [114].

Because of all these technical difficulties, as far as we know, only one research group was able to carry out a high-throughput characterization of PTMs in an organoid model, even though in a different context not associated with respiratory virus infection. In 2020, Qin and colleagues managed to perform a single-cell resolution analysis of PTMs in intestinal and colorectal cancer organoids, using mass cytometry technology [115] and published a protocol for multiplexed single-cell analysis of organoid signalling networks [112]. The technique is like flow cytometry, but the antibodies are conjugated with heavy metals, producing a recognizable fragmentation pattern in mass spectrometry instead of fluorophores recognized by a detector [116]. The heavy metal antibodies are used to (i) differentiate cells by cell type and (ii) identify specific PTMs in the organoid culture [112,115]. In this way, the authors managed to resolve cell-type-specific PTMs signalling networks in a heterogeneous culture model like intestinal organoids. This data has been produced by only one group and never replicated by others; thus, this approach deserves further investigation. Except for this report, as far as we know, no other studies on disease-associated PTMs using organoids have been published yet.

**Figure 2:**
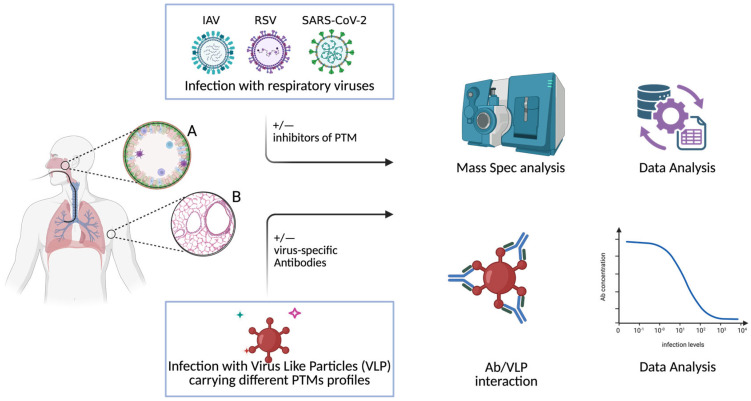
Experimental workflow to study PTM with organoids. A = upper airway system. B = lower way system. Details are described in the text.

Overall, with the attempt to provide a general overview of the workflow for experiments using the organoids, we can propose and discuss two ways (Figure 2). Organoids of the upper and lower respiratory tract can be used for both infection with replication-competent virus to study virus infection and viral-like particles with different PTM profiles to study only virus entry. In the first case, we can use inhibitors of the different PTMs to study how the virus fitness can be modulated in the presence or absence of specific PTMs, and the resulting viruses can be studied by mass spectrometry to identify the PTMs of the viruses with higher fitness. In the second case, we can first study if the presence or absence of specific PTM can affect the entry of the virus, besides also studying the impact of such modification on virus–antibody (Ab) interactions using a neutralization test.

## 6. Experimental Challenges in Detecting PTMs During Viral Infections and In Vitro Systems

In addition to the hurdles faced during the experimental settings of in vitro models to study PTMs in viral infections, a discussion must be carried out about the research methodology that can be used to detect PTMs, alongside their potentials and limitations [117,118,119].

Mass spectrometry (MS) is the gold standard for PTM discovery, enabling site-specific identification of glycosylation, phosphorylation, ubiquitination, acetylation, methylation, sulfation, and lipidation across thousands of proteins. Advances in glycoproteomics and the cutting-edge methods named “ubiquitin–remnant enrichment” improve detection of glycan microheterogeneity [45,120] and dynamic ubiquitin/acetylation patterns during infection [121]. Limitations include detection of low-abundance or transient PTMs, incomplete recovery of glycopeptides or peptides carrying lipid chains, and challenges in assigning labile modifications without specialized workflows [122].

PTM-specific antibodies complement MS by enabling high-throughput monitoring of modification dynamics in cells and tissues, but their utility is limited by off-target binding, cross-reactivity, and batch variability [123,124].

Functional mutagenesis and chemical labelling provide mechanistic insights by testing how PTM disruption affects viral entry, replication, protein stability, or immune recognition [36,44,125]. Metabolic labelling, lectin enrichment, and chemical tagging help dissect glycosylation and lipidation dynamics but may perturb physiology and often lack structural resolution [126,127].

Computational approaches are indispensable for interpreting high-throughput PTM datasets. A possible application of deep learning models such as DeepPhos, AttenPhos, and PhosIDN has been described for the prediction of phosphorylation sites [128,129,130,131]. Furthermore, network-based and knowledge-graph models, including NetKSA and KSFinder, can be used to uncover novel PTM networks that regulate the relationship between the kinases and their substrates [132,133]. Moreover, thanks to machine learning methods, it was possible to predict new methylation and acetylation sites for different classes of enzymes [134,135]. These bioinformatic and computational pipelines could also be used to guide experimental PTM discovery.

In conclusion, no single method fully captures the dynamic, context-dependent landscape of PTMs in viral infection. MS provides global maps and temporal dynamics, antibodies and mutagenesis validate functional relevance, and enzyme perturbation clarifies PTM hierarchies. Integrating PTM data with MS, transcriptomics, viral genetics, and structural information enables reconstruction of regulatory circuits that control viral entry, immune evasion, and host signalling. This multi-layered framework moves beyond descriptive PTM catalogues toward mechanistic models that guide hypothesis generation, prioritize functionally relevant modifications, and inform therapeutic and vaccine strategies [136,137].

## 7. Discussion

PTMs are chemical alterations that occur naturally after protein synthesis, playing a crucial role in regulating various cellular functions, but also in other contexts such as viral replication and host and immune evasion. In this review, we have reported the most relevant published works about PTMs related to three major respiratory viruses, IAV, RSV, and SARS-CoV-2, with a particular focus on the technological advancements to study them.

In vitro systems have significantly advanced the understanding of respiratory virus infections and PTMs, but several challenges remain. While 2D cell models are an easy-to-manage and cost-effective way to model respiratory tissues, they fail to represent tissue complexity and heterogeneity typically present in the respiratory system [82,138]. For instance, in the lungs, more than 40 different cell types are present [138] and bidimensional cultures have proven to be ineffective in modelling host–virus interactions and host immune responses against respiratory viruses [139]. For these reasons, in recent years, 3D respiratory organoid models have been developed to study PTMs in a more in vivo-like environment. While primary cells and organoids offer a higher physiological relevance, they are more challenging to produce and maintain compared to immortalized cell lines. Furthermore, the heterogeneity in these systems reduces reproducibility across laboratories, especially in more complex 3D cultures. Future models should incorporate immune cells to better study the interplay between infection, PTMs, and immune responses. Despite advances in in vitro modelling, translating findings related to PTMs to in vivo settings remains difficult. Overall, recently, the development of human airway organoids to study respiratory virus infections has gathered increased research interest, leading to the production of novel platforms suitable for disease modelling [114]. However, it is important to keep in mind that human airway organoids suffer from the same fallacies as other tissue organoids, including the heterogeneity of cellular populations present in the organoid and the high batch-to-batch variability, which could potentially limit the transfer of these results to preclinical models [87]. It is thus important that more efforts are spent on setting up this experimental model.

Another aspect that deserves further investigation is the impact of PTMs on the interaction between virus and host proteins. This topic could be discussed from at least two different angles: the interaction aiming at adaptation to different hosts and the interaction supporting the antiviral immunity. Examining PTMs and virus adaptation between different species and transmission events, it is important to note that PTMs can vary significantly across species due to evolutionary divergence and environmental factors [140]. Studies suggest that PTMs can be involved in viral adaptation, transmission, and pathogenicity, but data are limited, and further research is needed to better understand how species-specific PTMs influence the molecular mechanisms that underlie these processes [141,142,143].

**Figure 3:**
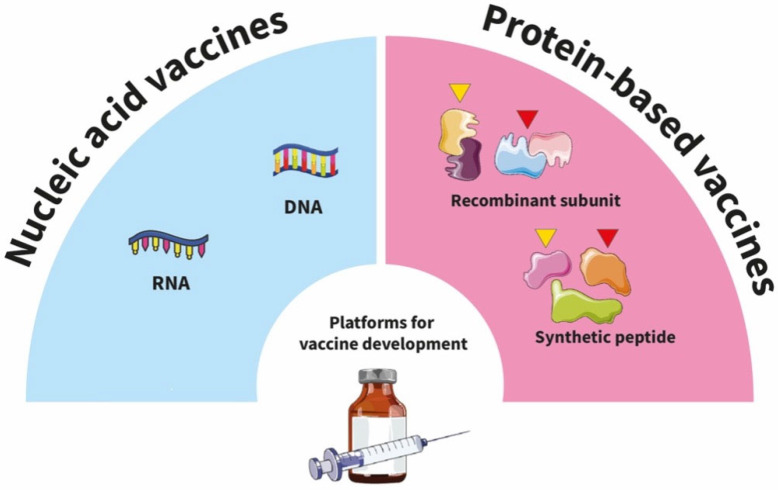
**Vaccine design and PTM.** Schematic overview showing the two major approaches to developing vaccines. Left/blue: nucleic acid-based vaccines (DNA or mRNA), where the antigen is produced inside host cells. Some PTMs may be added by the host machinery. Right/pink: protein-based vaccines, consisting of recombinant subunit proteins and synthetic peptides with (yellow flags) or without (red flags) PTMs.

The role of PTMs in antiviral immunity and vaccine development also deserves some discussion [38]. Despite the paucity of available data, we report studies showing that proteins expressed in mammalian systems, with glycosylation, have greater immunogenicity compared to those produced in bacterial systems [144]. For example, some previous studies [7] developed a glycan–protein conjugated vaccine for tick-borne encephalitis virus, where recombinant Bm86 and Bm95 proteins, expressed in Pichia pastoris, demonstrated enhanced immunogenicity compared to their non-glycosylated counterparts [145,146]. For vaccine development, one experimental approach could be to use the pseudovirus system to study the role of PTM in virus entry. A possible workflow could include the point mutagenesis of specific PTM sites to abrogate the PTM. In this way, the pseudo virus with the altered PTM profiles could be used to both study the impact of PTM on infectivity in different cell models and to investigate whether the PTM can have an impact on the binding of antibodies to virus surface proteins, affecting virus entry. In order to identify the PTM sites that need to be modified, a dedicated bioinformatic pipeline needs to be developed. Such novel data could help implement the current epitope database for vaccine development [147,148]. This aspect could be addressed by mutagenizing putative PTM target sites and studying their effect in 3D respiratory organoid infection models. This information could be relevant for the design of both innovative nucleic acid and protein-based vaccines (Figure 3).

## 8. Conclusions

To conclude, PTMs have been increasingly recognized as an important mechanism involved in cellular homeostasis and viral infection. Advances in cell culture techniques, organoid models, and proteomics have provided new insights into the complex molecular mechanisms driving viral infections. As these models continue to evolve, they hold great promise for identifying novel therapeutic targets, particularly those linked to PTMs, and improving our understanding of respiratory virus pathogenesis.

## Figures and Tables

**Table 1:** Major effects of PTM on viral infectivity and host response.

	IAV	RSV	SARS-CoV-2
**GLYCOSYLATION**	Enhances interaction with host receptors Influences viral tropism Can mediate immune evasion Might impair epitope exposure	Promotes infectivity in UTR	Increases viral replication Enhances viral proteins’ stability Affects tropism
**PHOSPHORYLATION**	Affects viral replication and assembly	Improves viral replication	Modulates viral replication and assembly Enhances infectivity in LTR
**UBIQUITINATION AND** **SUMOYLATION**	Modulation of viral stability and immune evasion	Related to the degradation of antiviral proteins Enhances immune evasion Increases viral replication	Inhibits viral replication
**S-PALMITOYLATION**	Promotes virion assembly and release	Enhances viral replication, particle formation, and infectivity	Aids in viral particle formation by stabilizing viral proteins

**Table 2:** Overview of current works using human respiratory organoid models to study influenza A virus (lAV). hESC = human embryonic stem cells.

Virus Infection Studies	Organoid Type	Model Applications	Reference
IAV	Human lung organoids derived from primary cells and inserted in a mechanical system mimicking human respiratory system	in vivo-like modelling of infection by H1N1 influenza virus identification of compounds as intranasal masks to avoid infection	(Hu et al., 2023) [92]
	Differentiated organoids generated from lung biopsies, protocol from (Sachs et al., 2019) [90]	investigation of differential infectivity of different influenza virus strains	(Zhou, J. et al. 2018) [93]
	Alveolar Type II lung organoids from hESC (line H9)	investigating molecular mechanisms of co-infection between Influenza A virus and SARS-CoV-2	(Kim et al., 2023) [94]
	Lung bud organoids from hESC (line H9)	preclinical drug testing of novel anti-influenza thiazolides	(Zhao et al., 2020) [95]
	Biopsy-derived human lung organoids	characterization of influenza strains tropism in human airway organoids	(Hui et al. 2018) [96]

**Table 3:** Overview of current works using human respiratory organoid models to study respiratory syncytial virus (RSV). iPSC = induced pluripotent stem cells; hPSC = human pluripotent stem cells: hESC = human embryonic stem cells; LC-MS = liquid chromatography coupled with mass spectrometry; PM = Particulate Mater, PM10 = Airborne particles ≤ 10 um in diameters.

Virus Infection Studies	Organoid Type	Model Applications	Reference
RSV	Human nose organoid obtained from nasal swabs/nasal wash	establishment of easy and non-invasive human nasal organoid model recapitulation of RSV pathology	(Rajan et al. 2021) [97]
	Human nose organoids obtained from nasal swabs/washes of adults and children	comparison of RSV replication, induction of cytokine response, cell injury and remodelling between young and adult nose organoids	(Aloisio et al. 2024) [85]
	iPSC-derived lung organoids	reproduction of architecture and transcriptional profile of feta lungs during 1st trimester of gestation characterization of RSV infection via LC-MS proteomics	(Harford et al. 2022) [98]
	hPSC-derived lung bud organoids	recapitulation of RSV pathology	(Chen et al. 2017) [99]
	Lung organoids from hESC (line H9)	investigation of PM10 and diesel PM effects on RSV-infected lung organoids	(Choi et al., 2022) [100]
	Lung organoids from non-small-cell lung cancer biopsies	recapitulation of RSV pathology in human cancerous lung model	(Sachs et al., 2019) [90]

**Table 4:** Overview of current works using human respiratory organoid models to study SARS-CoV-2. ACE2: angiotensin-converting enzyme 2; hESC = human embryonic stem cells; hPSC = human pluripotent stem cells.

Virus Infection Studies	Organoid Type	Model Applications	Reference
SARS-CoV2	hESC-derived lung organoids	identification of ACE2 regulatory mechanisms identification of therapeutic candidates	(Samuel et al., 2020) [101]
	hPSC-derived lung organoids	identification of cytokine and chemokine upregulation during infection high-throughput screening of FDA approved drugs	(Han et al., 2024) [102]
	hPSC-derived alveolar organoids	high-throughput screening of drugs	(Kim et al., 2021) [103]
	Human nose organoid obtained from nasal swabs/nasal wash	establishment of easy and non-invasive human nasal organoid model recapitulation of human pathological features	(Rajan et al. 2021) [97]
	Primary nasal epithelial cells-derived nasal organoid, containing ciliated, goblet and basal cells	study of viral attachment to cilia study of viral entry mechanisms identification of novel therapeutic candidates	(Wu et al., 2022) [104]
	Biopsy-derived bronchiolar and alveolar lung organoids	description of a protocol to obtain bronchi-specific and alveoli-specific organoids recapitulation of infection pathology	(Ekanger et al., 2022) [105]
	Lung bud tips organoid	production of renewable 2D bronchioalveolar model	(Lamers et al., 2021) [106]
	Alveolar Type II organoids from hESC (line H9)	investigating molecular mechanisms of co-infection between Influenza A virus and SARS-CoV-2	(Kim et al., 2023) [94]
	Bronchial organoids from normal human bronchial epithelial cells	cell response to viral infection by SARS-CoV2	(Sano et al., 2022) [107]

## Data Availability

No new data were created or analyzed in this study. Data sharing is not applicable to this article.

## References

[1] Girault J.-A., Preedy V.R. (2017). Chapter 13—Cocaine and Posttranslational Modifications of Neuronal Proteins. The Neuroscience of Cocaine.

[2] Zhong Q., Xiao X., Qiu Y., Xu Z., Chen C., Chong B., Zhao X., Hai S., Li S., An Z. (2023). Protein Posttranslational Modifications in Health and Diseases: Functions, Regulatory Mechanisms, and Therapeutic Implications. MedComm.

[3] Øye H., Lundekvam M., Caiella A., Hellesvik M., Arnesen T. (2025). Protein N-Terminal Modifications: Molecular Machineries and Biological Implications. Trends Biochem. Sci..

[4] Wang S., Osgood A.O., Chatterjee A. (2022). Uncovering Post-Translational Modification-Associated Protein–Protein Interactions. Curr. Opin. Struct. Biol..

[5] Zhang B., Schroeder F.C. (2025). Mechanisms of Metabolism-Coupled Protein Modifications. Nat. Chem. Biol..

[6] Deribe Y.L., Pawson T., Dikic I. (2010). Post-Translational Modifications in Signal Integration. Nat. Struct. Mol. Biol..

[7] Zhao Q., Zhou S., Lou W., Qian H., Xu Z. (2025). Crosstalk between O-GlcNAcylation and Phosphorylation in Metabolism: Regulation and Mechanism. Cell Death Differ..

[8] Kumar R., Mehta D., Mishra N., Nayak D., Sunil S. (2021). Role of Host-Mediated Post-Translational Modifications (PTMs) in RNA Virus Pathogenesis. Int. J. Mol. Sci..

[9] He M., Zhou X., Wang X. (2024). Glycosylation: Mechanisms, Biological Functions and Clinical Implications. Signal Transduct. Target. Ther..

[10] Lee J.M., Hammarén H.M., Savitski M.M., Baek S.H. (2023). Control of Protein Stability by Post-Translational Modifications. Nat. Commun..

[11] Schjoldager K.T., Narimatsu Y., Joshi H.J., Clausen H. (2020). Global View of Human Protein Glycosylation Pathways and Functions. Nat. Rev. Mol. Cell Biol..

[12] Contessa J.N., Bhojani M.S., Freeze H.H., Rehemtulla A., Lawrence T.S. (2008). Inhibition of N-Linked Glycosylation Disrupts Receptor Tyrosine Kinase Signaling in Tumor Cells. Cancer Res..

[13] Wang X., Gu J., Ihara H., Miyoshi E., Honke K., Taniguchi N. (2006). Core Fucosylation Regulates Epidermal Growth Factor Receptor-Mediated Intracellular Signaling. J. Biol. Chem..

[14] She Y.-M., Li X., Cyr T.D. (2019). Remarkable Structural Diversity of N-Glycan Sulfation on Influenza Vaccines. Anal. Chem..

[15] Walsh G., Jefferis R. (2006). Post-Translational Modifications in the Context of Therapeutic Proteins. Nat. Biotechnol..

[16] Ramazi S., Zahiri J. (2021). Post-Translational Modifications in Proteins: Resources, Tools and Prediction Methods. Database.

[17] Brazil D.P., Hemmings B.A. (2001). Ten Years of Protein Kinase B Signalling: A Hard Akt to Follow. Trends Biochem. Sci..

[18] Persad S., Attwell S., Gray V., Mawji N., Deng J.T., Leung D., Yan J., Sanghera J., Walsh M.P., Dedhar S. (2001). Regulation of Protein Kinase B/Akt-Serine 473 Phosphorylation by Integrin-Linked Kinase: CRITICAL ROLES FOR KINASE ACTIVITY AND AMINO ACIDS ARGININE 211 AND SERINE 343. J. Biol. Chem..

[19] Hunter T. (2014). The Genesis of Tyrosine Phosphorylation. Cold Spring Harb. Perspect. Biol..

[20] Wegmann S., Biernat J., Mandelkow E. (2021). A Current View on Tau Protein Phosphorylation in Alzheimer’s Disease. Curr. Opin. Neurobiol..

[21] Sharma C., Hamza A., Boyle E., Donu D., Cen Y. (2024). Post-Translational Modifications and Diabetes. Biomolecules.

[22] Montone R., Baruzzi A., Ferrarini F., Liboi E., Lievens P.M.-J., Romanelli M.G. (2017). Mutant FGFR3 Associated with SADDAN Disease Causes Cytoskeleton Disorganization through PLCγ1/Src-Mediated Paxillin Hyperphosphorylation. Int. J. Biochem. Amp Cell Biol..

[23] Singh V., Ram M., Kumar R., Prasad R., Roy B.K., Singh K.K. (2017). Phosphorylation: Implications in Cancer. Protein J..

[24] Park J., Cho J., Song E.J. (2020). Ubiquitin-Proteasome System (UPS) as a Target for Anticancer Treatment. Arch. Pharm. Res..

[25] Huang C.-H., Yang T.-T., Lin K.-I. (2024). Mechanisms and Functions of SUMOylation in Health and Disease: A Review Focusing on Immune Cells. J. Biomed. Sci..

[26] Emenike B., Nwajiobi O., Raj M. (2022). Covalent Chemical Tools for Profiling Post-Translational Modifications. Front. Chem..

[27] Yang Y., Bedford M.T. (2013). Protein Arginine Methyltransferases and Cancer. Nat. Rev. Cancer.

[28] Drazic A., Myklebust L.M., Ree R., Arnesen T. (2016). The World of Protein Acetylation. Biochim. Biophys. Acta.

[29] Dang F., Wei W. (2022). Targeting the Acetylation Signaling Pathway in Cancer Therapy. Semin. Cancer Biol..

[30] Chen B., Sun Y., Niu J., Jarugumilli G.K., Wu X. (2018). Protein Lipidation in Cell Signaling and Diseases: Function, Regulation, and Therapeutic Opportunities. Cell Chem. Biol..

[31] Yuan Y., Li P., Li J., Zhao Q., Chang Y., He X. (2024). Protein Lipidation in Health and Disease: Molecular Basis, Physiological Function and Pathological Implication. Signal Transduct. Target. Ther..

[32] Iwanaga T., Tsutsumi R., Noritake J., Fukata Y., Fukata M. (2009). Dynamic Protein Palmitoylation in Cellular Signaling. Prog. Lipid Res..

[33] Xiong X., Gao Y., Wang J., Wang H., Lou J., Bi Y., Yan Y., Li D., Song F. (2022). Palmitoyl Transferase FonPAT2-Catalyzed Palmitoylation of the FonAP-2 Complex Is Essential for Growth, Development, Stress Response, and Virulence in *Fusarium oxysporum* f. sp. *niveum*. Microbiol. Spectr..

[34] Mesquita F.S., Abrami L., Linder M.E., Bamji S.X., Dickinson B.C., van der Goot F.G. (2024). Mechanisms and Functions of Protein S-Acylation. Nat. Rev. Mol. Cell Biol..

[35] Lievens P.M.-J., Kuznetsova T., Kochlamazashvili G., Cesca F., Gorinski N., Galil D.A., Cherkas V., Ronkina N., Lafera J., Gaestel M. (2016). ZDHHC3 Tyrosine Phosphorylation Regulates Neural Cell Adhesion Molecule Palmitoylation. Mol. Cell. Biol..

[36] Busquets-Hernández C., Triola G. (2021). Palmitoylation as a Key Regulator of Ras Localization and Function. Front. Mol. Biosci..

[37] Fung T.S., Liu D.X. (2018). Post-Translational Modifications of Coronavirus Proteins: Roles and Function. Future Virol..

[38] Ojha R., Prajapati V.K. (2021). Cognizance of Posttranslational Modifications in Vaccines: A Way to Enhanced Immunogenicity. J. Cell. Physiol..

[39] Zarling A.L., Polefrone J.M., Evans A.M., Mikesh L.M., Shabanowitz J., Lewis S.T., Engelhard V.H., Hunt D.F. (2006). Identification of Class I MHC-Associated Phosphopeptides as Targets for Cancer Immunotherapy. Proc. Natl. Acad. Sci. USA.

[40] Zhu G., Tong N., Zhu Y., Wang L., Wang Q. (2025). The Crosstalk between SUMOylation and Immune System in Host-Pathogen Interactions. Crit. Rev. Microbiol..

[41] Jeng M.Y., Ali I., Ott M. (2015). Manipulation of the Host Protein Acetylation Network by Human Immunodeficiency Virus Type 1. Crit. Rev. Biochem. Mol. Biol..

[42] Manzoor R., Igarashi M., Takada A. (2017). Influenza A Virus M2 Protein: Roles from Ingress to Egress. Int. J. Mol. Sci..

[43] Ohol Y.M., Wang Z., Kemble G., Duke G. (2015). Direct Inhibition of Cellular Fatty Acid Synthase Impairs Replication of Respiratory Syncytial Virus and Other Respiratory Viruses. PLoS ONE.

[44] Puthenveetil R., Lun C.M., Murphy R.E., Healy L.B., Vilmen G., Christenson E.T., Freed E.O., Banerjee A. (2021). S-Acylation of SARS-CoV-2 Spike Protein: Mechanistic Dissection, in Vitro Reconstitution and Role in Viral Infectivity. J. Biol. Chem..

[45] Li X., Shen L., Xu Z., Liu W., Li A., Xu J. (2022). Protein Palmitoylation Modification During Viral Infection and Detection Methods of Palmitoylated Proteins. Front. Cell. Infect. Microbiol..

[46] Locatelli M., Faure-Dupuy S. (2023). Virus Hijacking of Host Epigenetic Machinery to Impair Immune Response. J. Virol..

[47] Hu J., Zhang L., Liu X. (2020). Role of Post-Translational Modifications in Influenza A Virus Life Cycle and Host Innate Immune Response. Front. Microbiol..

[48] Dou D., Revol R., Östbye H., Wang H., Daniels R. (2018). Influenza A Virus Cell Entry, Replication, Virion Assembly and Movement. Front. Immunol..

[49] Richard M., van den Brand J.M.A., Bestebroer T.M., Lexmond P., de Meulder D., Fouchier R.A.M., Lowen A.C., Herfst S. (2020). Influenza A Viruses Are Transmitted via the Air from the Nasal Respiratory Epithelium of Ferrets. Nat. Commun..

[50] CDC About Common Cold. https://www.cdc.gov/common-cold/about/index.html.

[51] Hook J.L., Bhattacharya J. (2024). The Pathogenesis of Influenza in Intact Alveoli: Virion Endocytosis and Its Effects on the Lung’s Air-Blood Barrier. Front. Immunol..

[52] Sumitomo T., Kawabata S. (2024). Respiratory Tract Barrier Dysfunction in Viral-Bacterial Co-Infection Cases. Jpn. Dent. Sci. Rev..

[53] York I.A., Stevens J., Alymova I.V. (2019). Influenza Virus N-Linked Glycosylation and Innate Immunity. Biosci. Rep..

[54] Tsai M., Koch D., Forchione A., Farkas L., El-Mergawy R., Londino J.D., Mallampalli R.K. (2025). Ubiquitin E3 Ligase MARCH10 Targets Influenza Hemagglutinin for Ubiquitination. Cell. Signal..

[55] Park E.-S., Dezhbord M., Lee A.R., Kim K.-H. (2022). The Roles of Ubiquitination in Pathogenesis of Influenza Virus Infection. Int. J. Mol. Sci..

[56] Wu C.-Y., Jeng K.-S., Lai M.M.-C. (2011). The SUMOylation of Matrix Protein M1 Modulates the Assembly and Morphogenesis of Influenza A Virus. J. Virol..

[57] Cong J., Wang T., Hahm B., Xia C. (2025). Positive Regulation of Cellular Proteins by Influenza Virus for Productive Infection. Int. J. Mol. Sci..

[58] Bergeron H.C., Tripp R.A. (2022). RSV Replication, Transmission, and Disease Are Influenced by the RSV G Protein. Viruses.

[59] Jain H., Schweitzer J.W., Justice N.A. (2024). Respiratory Syncytial Virus Infection in Children. StatPearls.

[60] Griffiths C., Drews S.J., Marchant D.J. (2017). Respiratory Syncytial Virus: Infection, Detection, and New Options for Prevention and Treatment. Clin. Microbiol. Rev..

[61] Feldman S.A., Audet S., Beeler J.A. (2000). The Fusion Glycoprotein of Human Respiratory Syncytial Virus Facilitates Virus Attachment and Infectivity via an Interaction with Cellular Heparan Sulfate. J. Virol..

[62] King T., Mejias A., Ramilo O., Peeples M.E. (2021). The Larger Attachment Glycoprotein of Respiratory Syncytial Virus Produced in Primary Human Bronchial Epithelial Cultures Reduces Infectivity for Cell Lines. PLoS Pathog..

[63] Okura T., Takahashi T., Kameya T., Mizukoshi F., Nakai Y., Kakizaki M., Nishi M., Otsuki N., Kimura H., Miyakawa K. (2024). MARCH8 Restricts RSV Replication by Promoting Cellular Apoptosis Through Ubiquitin-Mediated Proteolysis of Viral SH Protein. Viruses.

[64] Basse V., Wang Y., Rodrigues-Machado C., Henry C., Richard C.-A., Leyrat C., Galloux M. (2025). Regulation of Respiratory Syncytial Virus Nucleoprotein Oligomerization by Phosphorylation. J. Biol. Chem..

[65] Cheng N., Liu M., Li W., Sun B., Liu D., Wang G., Shi J., Li L. (2023). Protein Post-Translational Modification in SARS-CoV-2 and Host Interaction. Front. Immunol..

[66] Noettger S., Zech F., Nchioua R., Pastorio C., Jung C., Jacob T., Stenger S., Kirchhoff F. (2025). Role of N-Linked Glycosylation Sites in Human ACE2 in SARS-CoV-2 and hCoV-NL63 Infection. J. Virol..

[67] Xiao Y., Chang L., Ji H., Sun H., Song S., Feng K., Nuermaimaiti A., Halemubieke S., Mei L., Lu Z. (2023). Posttranslational Modifications of ACE2 Protein: Implications for SARS-CoV-2 Infection and Beyond. J. Med. Virol..

[68] Lauster D., Osterrieder K., Haag R., Ballauff M., Herrmann A. (2023). Respiratory Viruses Interacting with Cells: The Importance of Electrostatics. Front. Microbiol..

[69] Trypsteen W., Van Cleemput J., van Snippenberg W., Gerlo S., Vandekerckhove L. (2020). On the Whereabouts of SARS-CoV-2 in the Human Body: A Systematic Review. PLoS Pathog..

[70] Xiao Y., Gao M., Mo X., Lang J., Wang Z., Ma Z., Yang M., Tang B., Liu D., He H. (2025). Mechanisms and Research Methods of Protein Modification in Virus Entry. Appl. Biochem. Biotechnol..

[71] Gallo O., Locatello L.G., Mazzoni A., Novelli L., Annunziato F. (2021). The Central Role of the Nasal Microenvironment in the Transmission, Modulation, and Clinical Progression of SARS-CoV-2 Infection. Mucosal Immunol..

[72] Xia B., Shen X., He Y., Pan X., Liu F.-L., Wang Y., Yang F., Fang S., Wu Y., Duan Z. (2021). SARS-CoV-2 Envelope Protein Causes Acute Respiratory Distress Syndrome (ARDS)-like Pathological Damages and Constitutes an Antiviral Target. Cell Res..

[73] Gonzalez-Orozco M., Tseng H., Hage A., Xia H., Behera P., Afreen K., Peñaflor-Tellez Y., Giraldo M.I., Huante M., Puebla-Clark L. (2024). TRIM7 Ubiquitinates SARS-CoV-2 Membrane Protein to Limit Apoptosis and Viral Replication. Nat. Commun..

[74] Wang Z., Qiu M., Ji Y., Chai K., Liu C., Xu F., Guo F., Tan J., Liu R., Qiao W. (2024). Palmitoylation of SARS-CoV-2 Envelope Protein Is Central to Virus Particle Formation. J. Virol..

[75] Tanneti N.S., Patel A.K., Tan L.H., Marques A.D., Perera R.A.P.M., Sherrill-Mix S., Kelly B.J., Renner D.M., Collman R.G., Rodino K. (2024). Comparison of SARS-CoV-2 Variants of Concern in Primary Human Nasal Cultures Demonstrates Delta as Most Cytopathic and Omicron as Fastest Replicating. mBio.

[76] Ramadan A.A., Mayilsamy K., McGill A.R., Ghosh A., Giulianotti M.A., Donow H.M., Mohapatra S.S., Mohapatra S., Chandran B., Deschenes R.J. (2022). Identification of SARS-CoV-2 Spike Palmitoylation Inhibitors That Results in Release of Attenuated Virus with Reduced Infectivity. Viruses.

[77] Vimalajeewa D., Balasubramaniam S., Berry D.P., Barry G. (2022). Virus Particle Propagation and Infectivity along the Respiratory Tract and a Case Study for SARS-CoV-2. Sci. Rep..

[78] Zhang Y., Zhu J., Li Y., Bradley K.C., Cao J., Chen H., Jin M., Zhou H. (2013). Glycosylation on Hemagglutinin Affects the Virulence and Pathogenicity of Pandemic H1N1/2009 Influenza A Virus in Mice. PLoS ONE.

[79] Valerdi K.M., Hage A., van Tol S., Rajsbaum R., Giraldo M.I. (2021). The Role of the Host Ubiquitin System in Promoting Replication of Emergent Viruses. Viruses.

[80] Zheng X., Wang L., Zhang Z., Tang H. (2023). The Emerging Roles of SUMOylation in Pulmonary Diseases. Mol. Med..

[81] Bajaj S., Sharma N. (2021). Different Cell Lines for SARS-CoV-2. Coronavirus Disease-19 (COVID-19): A Perspective of New Scenario.

[82] Joo H., Min S., Cho S.-W. (2024). Advanced Lung Organoids for Respiratory System and Pulmonary Disease Modeling. J. Tissue Eng..

[83] Leung C., Wadsworth S.J., Yang S.J., Dorscheid D.R. (2020). Structural and Functional Variations in Human Bronchial Epithelial Cells Cultured in Air-Liquid Interface Using Different Growth Media. Am. J. Physiol. Lung Cell. Mol. Physiol..

[84] Barron S.L., Saez J., Owens R.M. (2021). In Vitro Models for Studying Respiratory Host–Pathogen Interactions. Adv. Biol..

[85] Aloisio G.M., Nagaraj D., Murray A.M., Schultz E.M., McBride T., Aideyan L., Nicholson E.G., Henke D., Ferlic-Stark L., Rajan A. (2024). Infant-Derived Human Nasal Organoids Exhibit Relatively Increased Susceptibility, Epithelial Responses, and Cytotoxicity during RSV Infection. J. Infect..

[86] Petpiroon N., Netkueakul W., Sukrak K., Wang C., Liang Y., Wang M., Liu Y., Li Q., Kamran R., Naruse K. (2023). Development of Lung Tissue Models and Their Applications. Life Sci..

[87] Bombieri C., Corsi A., Trabetti E., Ruggiero A., Marchetto G., Vattemi G., Valenti M.T., Zipeto D., Romanelli M.G. (2024). Advanced Cellular Models for Rare Disease Study: Exploring Neural, Muscle and Skeletal Organoids. Int. J. Mol. Sci..

[88] Lancaster M.A., Knoblich J.A. (2014). Organogenesis in a Dish: Modeling Development and Disease Using Organoid Technologies. Science.

[89] Yamamoto Y., Ochiya T. (2017). Epithelial Stem Cell Culture: Modeling Human Disease and Applications for Regenerative Medicine. Inflamm. Regen..

[90] Sachs N., Papaspyropoulos A., Zomer-van Ommen D.D., Heo I., Böttinger L., Klay D., Weeber F., Huelsz-Prince G., Iakobachvili N., Amatngalim G.D. (2019). Long-term Expanding Human Airway Organoids for Disease Modeling. EMBO J..

[91] Dye B.R., Hill D.R., Ferguson M.A., Tsai Y.-H., Nagy M.S., Dyal R., Wells J.M., Mayhew C.N., Nattiv R., Klein O.D. (2015). In Vitro Generation of Human Pluripotent Stem Cell Derived Lung Organoids. eLife.

[92] Hu X., Wang S., Fu S., Qin M., Lyu C., Ding Z., Wang Y., Wang Y., Wang D., Zhu L. (2023). Intranasal mask for protecting the respiratory tract against viral aerosols. Nat. Commun..

[93] Zhou J., Li C., Sachs N., Chiu M.C., Wong B.H.-Y., Chu H., Poon V.K.-M., Wang D., Zhao X., Wen L. (2018). Differentiated Human Airway Organoids to Assess Infectivity of Emerging Influenza Virus. Proc. Natl. Acad. Sci. USA.

[94] Kim M.J., Kim S., Kim H., Gil D., Han H.-J., Thimmulappa R.K., Choi J.-H., Kim J.-H. (2023). Reciprocal enhancement of SARS-CoV-2 and influenza virus replication in human pluripotent stem cell-derived lung organoids. Emerg. Microbes Infect..

[95] Zhao L., Yan Y., Dai Q., Li X., Xu K., Zou G., Yang K., Li W., Guo X., Yang J. (2020). Development of Novel Anti-influenza Thiazolides with Relatively Broad-Spectrum Antiviral Potentials. Antimicrob. Agents Chemother..

[96] Hui K.P.Y., Ching R.H.H., Chan S.K.H., Nicholls J.M., Sachs N., Clevers H., Peiris J.S.M., Chan M.C.W. (2018). Tropism, Replication Competence, and Innate Immune Responses of Influenza Virus: An Analysis of Human Airway Organoids and Ex-Vivo Bronchus Cultures. Lancet Respir. Med..

[97] Rajan A., Weaver A.M., Aloisio G.M., Jelinski J., Johnson H.L., Venable S.F., McBride T., Aideyan L., Piedra F.-A., Ye X. (2021). The Human Nose Organoid Respiratory Virus Model: An Ex Vivo Human Challenge Model to Study Respiratory Syncytial Virus (RSV) and Severe Acute Respiratory Syndrome Coronavirus 2 (SARS-CoV-2) Pathogenesis and Evaluate Therapeutics. mBio.

[98] Harford T.J., Rezaee F., Dye B.R., Fan J., Spence J.R., Piedimonte G. (2022). RSV-Induced Changes in a 3-Dimensional Organoid Model of Human Fetal Lungs. PLoS ONE.

[99] Chen Y.-W., Huang S.X., de Carvalho A.L.R.T., Ho S.-H., Islam M.N., Volpi S., Notarangelo L.D., Ciancanelli M., Casanova J.-L., Bhattacharya J. (2017). A Three-Dimensional Model of Human Lung Development and Disease from Pluripotent Stem Cells. Nat. Cell Biol..

[100] Choi S., Kim E.-M., Kim S.-Y., Choi Y., Choi S., Cho N., Park H.-J., Kim K.K. (2022). Particulate matter exposure exacerbates cellular damage by increasing stress granule formation in respiratory syncytial virus-infected human lung organoids. Environ. Pollut..

[101] Samuel R.M., Majd H., Richter M.N., Ghazizadeh Z., Zekavat S.M., Navickas A., Ramirez J.T., Asgharian H., Simoneau C.R., Bonser L.R. (2020). Androgen Signaling Regulates SARS-CoV-2 Receptor Levels and Is Associated with Severe COVID-19 Symptoms in Men. Cell Stem Cell.

[102] Han Y., Duan X., Yang L., Nilsson-Payant B.E., Wang P., Duan F., Tang X., Yaron T.M., Zhang T., Uhl S. (2021). Identification of SARS-CoV-2 inhibitors using lung and colonic organoids. Nature.

[103] Kim J.-H., An G.H., Kim J.-Y., Rasaei R., Kim W.J., Jin X., Woo D.-H., Han C., Yang S.-R., Kim J.-H. (2021). Human pluripotent stem cell-derived alveolar organoids for modeling pulmonary fibrosis and drug testing. Cell Death Discov..

[104] Wu C.-T., Lidsky P.V., Xiao Y., Cheng R., Lee I.T., Nakayama T., Jiang S., He W., Demeter J., Knight M.G. (2022). SARS-CoV-2 replication in airway epithelia requires motile cilia and microvillar reprogramming. Cell.

[105] Ekanger C.T., Zhou F., Bohan D., Lotsberg M.L., Ramnefjell M., Hoareau L., Røsland G.V., Lu N., Aanerud M., Gärtner F. (2022). Human Organotypic Airway and Lung Organoid Cells of Bronchiolar and Alveolar Differentiation Are Permissive to Infection by Influenza and SARS-CoV-2 Respiratory Virus. Front. Cell. Infect. Microbiol..

[106] Lamers M.M., van der Vaart J., Knoops K., Riesebosch S., Breugem T.I., Mykytyn A.Z., Beumer J., Schipper D., Bezstarosti K., Koopman C.D. (2021). An organoid-derived bronchioalveolar model for SARS-CoV-2 infection of human alveolar type II-like cells. EMBO J..

[107] Sano E., Suzuki T., Hashimoto R., Itoh Y., Sakamoto A., Sakai Y., Saito A., Okuzaki D., Motooka D., Muramoto Y. (2022). Cell response analysis in SARS-CoV-2 infected bronchial organoids. Commun. Biol..

[108] Duan X., Tang X., Nair M.S., Zhang T., Qiu Y., Zhang W., Wang P., Huang Y., Xiang J., Wang H. (2021). An Airway Organoid-Based Screen Identifies a Role for the HIF1α-Glycolysis Axis in SARS-CoV-2 Infection. Cell Rep..

[109] Kim S.K., Sung E., Lim K. (2024). Recent Advances and Applications of Human Lung Alveolar Organoids. Mol. Cells.

[110] Borchers A.T., Chang C., Gershwin M.E., Gershwin L.J. (2013). Respiratory Syncytial Virus—A Comprehensive Review. Clin. Rev. Allergy Immunol..

[111] Soni A., Kabra S.K., Lodha R. (2023). Respiratory Syncytial Virus Infection: An Update. Indian J. Pediatr..

[112] Sufi J., Qin X., Rodriguez F.C., Bu Y.J., Vlckova P., Zapatero M.R., Nitz M., Tape C.J. (2021). Multiplexed single-cell analysis of organoid signaling networks. Nat. Protoc..

[113] Simmons A.J., Banerjee A., McKinley E.T., Scurrah C.R., Herring C.A., Gewin L.S., Masuzaki R., Karp S.J., Franklin J.L., Gerdes M.J. (2015). Cytometry-Based Single-Cell Analysis of Intact Epithelial Signaling Reveals MAPK Activation Divergent from TNF-α-Induced Apoptosis In Vivo. Mol. Syst. Biol..

[114] Dobric A., Tape C.J. (2025). High-Dimensional Signalling Analysis of Organoids. Curr. Opin. Cell Biol..

[115] Qin X., Sufi J., Vlckova P., Kyriakidou P., Acton S.E., Li V.S.W., Nitz M., Tape C.J. (2020). Cell-Type-Specific Signaling Networks in Heterocellular Organoids. Nat. Methods.

[116] Watson E.C.R., Baker W., Ahern D., Loi D., Cribbs A.P., Oppermann U. (2023). Mass Cytometry as a Tool in Target Validation and Drug Discovery. Methods Enzymol..

[117] Liang B., Zhu Y., Shi W., Ni C., Tan B., Tang S. (2023). SARS-CoV-2 Spike Protein Post-Translational Modification Landscape and Its Impact on Protein Structure and Function via Computational Prediction. Res. Wash. DC.

[118] Bludau I., Aebersold R. (2020). Proteomic and Interactomic Insights into the Molecular Basis of Cell Functional Diversity. Nat. Rev. Mol. Cell Biol..

[119] Madahar V., Dang R., Zhang Q., Liu C., Rodgers V.G.J., Liao J. (2023). Human Post-Translational SUMOylation Modification of SARS-CoV-2 Nucleocapsid Protein Enhances Its Interaction Affinity with Itself and Plays a Critical Role in Its Nuclear Translocation. Viruses.

[120] Shajahan A., Pepi L.E., Kumar B., Murray N.B., Azadi P. (2023). Site Specific N- and O-Glycosylation Mapping of the Spike Proteins of SARS-CoV-2 Variants of Concern. Sci. Rep..

[121] Zhang T., Wang Y., Sun Y., Song M., Pang J., Wang M., Zhang Z., Yang P., Chen Y., Qi X. (2024). Proteome, Lysine Acetylome, and Succinylome Identify Posttranslational Modification of STAT1 as a Novel Drug Target in Silicosis. Mol. Cell. Proteom..

[122] Liu J.-F., Peng W.-J., Wu Y., Yang Y.-H., Wu S.-F., Liu D.-P., Liu J.-N., Yang J.-T. (2023). Proteomic and Phosphoproteomic Characteristics of the Cortex, Hippocampus, Thalamus, Lung, and Kidney in COVID-19-Infected Female K18-hACE2 Mice. eBioMedicine.

[123] Riso M., Shah R.N., Koide A., Ruthenburg A.J., Koide S., Hattori T. (2025). Binding Mode-Guided Development of High-Performance Antibodies Targeting Site-Specific Posttranslational Modifications. Proc. Natl. Acad. Sci. USA.

[124] Mann G., Sulkshane P., Sadhu P., Ziv T., Glickman M.H., Brik A. (2022). Antibody for Serine 65 Phosphorylated Ubiquitin Identifies PLK1-Mediated Phosphorylation of Mitotic Proteins and APC1. Molecules.

[125] Watanabe Y., Allen J.D., Wrapp D., McLellan J.S., Crispin M. (2020). Site-Specific Glycan Analysis of the SARS-CoV-2 Spike. Science.

[126] Merz N., Schilling K., Thomas D., Hahnefeld L., Grösch S. (2025). Cell Labeling with 15-YNE Is Useful for Tracking Protein Palmitoylation and Metabolic Lipid Flux in the Same Sample. Molecules.

[127] Townsend C.A., Petropavlovskiy A.A., Kogut J.A., Church A.M., Sanders S.S. (2024). Protocol to Identify S-Acylated Proteins in Hippocampal Neurons Using ω-Alkynyl Fatty Acid Analogs and Click Chemistry. STAR Protoc..

[128] Luo F., Wang M., Liu Y., Zhao X.-M., Li A. (2019). DeepPhos: Prediction of Protein Phosphorylation Sites with Deep Learning. Bioinformatics.

[129] Yang H., Wang M., Liu X., Zhao X.-M., Li A. (2021). PhosIDN: An Integrated Deep Neural Network for Improving Protein Phosphorylation Site Prediction by Combining Sequence and Protein–Protein Interaction Information. Bioinformatics.

[130] Song T., Yang Q., Qu P., Qiao L., Wang X. (2024). Attenphos: General Phosphorylation Site Prediction Model Based on Attention Mechanism. Int. J. Mol. Sci..

[131] Chang X., Zhu Y., Chen Y., Li L. (2024). DeepNphos: A Deep-Learning Architecture for Prediction of N-Phosphorylation Sites. Comput. Biol. Med..

[132] Ayati M., Yilmaz S., Blasco Tavares Pereira Lopes F., Chance M., Koyuturk M. (2023). Prediction of Kinase-Substrate Associations Using the Functional Landscape of Kinases and Phosphorylation Sites. Pac. Symp. Biocomput. Pac. Symp. Biocomput..

[133] Anandakrishnan M., Ross K.E., Chen C., Shanker V., Cowart J., Wu C.H. (2023). KSFinder—A Knowledge Graph Model for Link Prediction of Novel Phosphorylated Substrates of Kinases. PeerJ.

[134] Smith B.C., Settles B., Hallows W.C., Craven M.W., Denu J.M. (2011). SIRT3 Substrate Specificity Determined by Peptide Arrays and Machine Learning. ACS Chem. Biol..

[135] Ridgeway N.H., Chopra A., Lukinović V., Feldman M., Charih F., Levy D., Green J.R., Biggar K.K. (2025). Machine Learning-Driven Prediction of Substrates for Enzymes Introducing or Removing Protein Post-Translational Modifications. Commun. Chem..

[136] Bludau I., Willems S., Zeng W.-F., Strauss M.T., Hansen F.M., Tanzer M.C., Karayel O., Schulman B.A., Mann M. (2022). The Structural Context of Posttranslational Modifications at a Proteome-Wide Scale. PLoS Biol..

[137] Leutert M., Entwisle S.W., Villén J. (2021). Decoding Post-Translational Modification Crosstalk with Proteomics. Mol. Cell. Proteom..

[138] Sun X., Perl A.-K., Li R., Bell S.M., Sajti E., Kalinichenko V.V., Kalin T.V., Misra R.S., Deshmukh H., Clair G. (2022). A Census of the Lung: CellCards from LungMAP. Dev. Cell.

[139] Edwards C.E., Tata A., Baric R.S. (2022). Human Lung Organoids as a Model for Respiratory Virus Replication and Countermeasure Performance in Human Hosts. Transl. Res..

[140] Beltrao P., Bork P., Krogan N.J., van Noort V. (2013). Evolution and Functional Cross-talk of Protein Post-translational Modifications. Mol. Syst. Biol..

[141] Boytz R., Laurent-Rolle M. (2024). Balancing Acts: The Posttranslational Modification Tightrope of Flavivirus Replication. PLoS Pathog..

[142] Li X., Kabza A., Ives A.N., Thiel J., Waters K.M., Qian W.-J., Sims A.C., Zhang T. (2025). Proteome-Wide Characterization of PTMs Reveals Host Cell Responses to Viral Infection and Identifies Putative Antiviral Drug Targets. Front. Immunol..

[143] Schmidt H.M., Horner S.M. (2025). Towards a Universal Translator: Decoding the PTMs That Regulate Orthoflavivirus Infection. Viruses.

[144] Nascimento I.P., Leite L.C.C. (2012). Recombinant Vaccines and the Development of New Vaccine Strategies. Braz. J. Med. Biol. Res..

[145] de la Fuente J., Canales M., Kocan K.M. (2006). The Importance of Protein Glycosylation in Development of Novel Tick Vaccine Strategies. Parasite Immunol..

[146] Nuttall P.A., Trimnell A.R., Kazimirova M., Labuda M. (2006). Exposed and Concealed Antigens as Vaccine Targets for Controlling Ticks and Tick-borne Diseases. Parasite Immunol..

[147] Flender D., Vilenne F., Adams C., Boonen K., Valkenborg D., Baggerman G. (2025). Exploring the Dynamic Landscape of Immunopeptidomics: Unravelling Posttranslational Modifications and Navigating Bioinformatics Terrain. Mass Spectrom. Rev..

[148] Basmenj E.R., Pajhouh S.R., Ebrahimi Fallah A., Naijian R., Rahimi E., Atighy H., Ghiabi S., Ghiabi S. (2025). Computational Epitope-Based Vaccine Design with Bioinformatics Approach; a Review. Heliyon.

